# POSEA: A novel algorithm to evaluate the performance of multi-object instance image segmentation

**DOI:** 10.1371/journal.pone.0283692

**Published:** 2023-03-29

**Authors:** Nianchao Wang, Linghao Hu, Alex J. Walsh

**Affiliations:** Texas A&M University, TAMU, College Station, Texas, United States of America; BMS Institute of Technology and Management, INDIA

## Abstract

Many techniques and software packages have been developed to segment individual cells within microscopy images, necessitating a robust method to evaluate images segmented into a large number of unique objects. Currently, segmented images are often compared with ground-truth images at a pixel level; however, this standard pixel-level approach fails to compute errors due to pixels incorrectly assigned to adjacent objects. Here, we define a per-object segmentation evaluation algorithm (POSEA) that calculates segmentation accuracy metrics for each segmented object relative to a ground truth segmented image. To demonstrate the performance of POSEA, precision, recall, and f-measure metrics are computed and compared with the standard pixel-level evaluation for simulated images and segmented fluorescence microscopy images of three different cell samples. POSEA yields lower accuracy metrics than the standard pixel-level evaluation due to correct accounting of misclassified pixels of adjacent objects. Therefore, POSEA provides accurate evaluation metrics for objects with pixels incorrectly assigned to adjacent objects and is robust for use across a variety of applications that require evaluation of the segmentation of unique adjacent objects.

## Introduction

Altered cellular-level heterogeneity within tissues is a characteristic of many diseases, including autoimmune disease [1], fibrotic skin disease [2], lysosomal storage disease [3], and cancer [4]. Tumors are complex tissues comprised of not only cancer cells, but also vasculature, immune cells, and stromal cells. High levels of intratumoral heterogeneity predispose patients to inferior clinical outcomes since resistance can emerge as a result of drug-tolerant populations [5]. Therefore, identification and quantification of cellular-level heterogeneity are important for the study of tissue pathologies and therapies. However, the assessment of cellular heterogeneity remains challenging. Traditional biochemical assays such as western blot [6], mRNA analysis [7], and oxygen consumption assays (i.e. Seahorse assay [8]) typically require the pooling of substrates from thousands of cells and do not provide single-cell information. Alternatively, single-cell assessment technologies such as flow cytometry and single-cell RNA sequencing require a homogenized cell suspension, which destroys the spatial integrity of the sample. Traditional biochemistry techniques also often require cell permeabilization for labeling with exogenous contrast agents, which limits *in vivo* and dynamic or time-course studies.

Fluorescence microscopy can be used for cell heterogeneity analysis if the images are segmented and analyzed at a single-cell level. Fluorescence imaging of the endogenous metabolic cofactors reduced nicotinamide adenine dinucleotide (NAD(P)H) and flavin adenine dinucleotide (FAD) enables nondestructive evaluation of cellular metabolism [9–11]. Single-cell segmentation of fluorescence images has been used to assess immune cell heterogeneity [12–14], cancer heterogeneity [15–18], cellular heterogeneity in response to treatment [19–22] and spatial intratumoral heterogeneity [23,24]. Heterogeneity analysis of fluorescence images requires instance segmentation of the image into individual cells. Multiple solutions for instance segmentation of fluorescence images exist. These algorithms are often tailored to a specific image dataset and include a series of traditional image processing steps, such as intensity-based threshold and watershed [25–27], or use machine learning techniques, such as convolutional neural networks, to classify pixels for image segmentation [28–31]. Many software packages are available for fluorescence image segmentation including ImageJ, CellProfiler, Ilastik, and Imaris [32,33].

Due to a large number of available image segmentation tools, it is important to robustly evaluate segmentation results. A variety of methods can be used to evaluate image segmentation. Traditional subjective methods use human evaluators to provide qualitative assessment scores of the segmentation results, but subjective evaluation lacks consistency and is time-consuming [34]. Objective methods quantify the segmentation results relative to a ground truth segmented image [35]. Evaluation metrics such as Precision and Dice Score are based on true positive (TP), false positive (FP), true negative (TN), and false negative (FN) classifications of pixels in the segmented image determined relative to the ground truth image [36]. While the assignment of pixels is relatively simple for semantic segmentation, where pixels are typically assigned to one of two classes, object or background, pixel-level assessment is not accurate for instance segmentation where a pixel must not only be correctly labeled as object but also attributed to the correct object. Often fluorescence signals within cells are localized to compartments such as the cytoplasm and downstream analysis of cell heterogeneity requires not only high accuracy of cell identification but also accurate pixel assignments to ensure the data is not confounded by background pixels.

Metrics can also evaluate instance segmentation performance. Several object-based evaluation algorithms have been demonstrated to evaluate over-segmentation in multispectral satellite images, which present multiple object classes of generally disparate objects [37,38]. Additionally, the COCO metrics are commonly used evaluation metrics for instance segmentation and include 12 metrics in four categories: Average Precision (AP), AP across scales, Average Recall (AR), and AR across scales [39]. However, these evaluation techniques provide object-level metrics and are not directly applicable to evaluate the accuracy of both object detection and the within object pixel accuracy. A robust algorithm for the accurate evaluation of instance cell segmentation results at both an object and pixel level remains unexplored for images with a large number of adjacent objects.

In this paper, we define and demonstrate the performance of a supervised per-object segmentation evaluation algorithm (POSEA). Traditionally, segmented images are often compared at a pixel level without accounting for object-specificity [34]; however, a standard pixel-level approach fails to compute errors due to pixels correctly attributed to the object class but incorrectly assigned to an adjacent object. POSEA addresses this inaccuracy problem by extracting each object from the ground truth segmented image, matching it with the colocalized segmented object, and assigning each pixel within the segmented image as true positive (TP), true negative (TN), false positive (FP), or false negative (FN) for computation of traditional performance metrics such as Precision, Recall, and F-measure. POSEA was tested on simulated binary and grayscale images and was used to evaluate the performance of CellProfiler segmentation results of autofluorescence images of three different cell samples of varying segmentation complexity. Based on the results, POSEA is advantageous for the supervised evaluation of segmented images with a large number of unique, adjacent objects.

## Materials and methods

### POSEA

#### Traditional pixel-level evaluation

**Fig 1:**
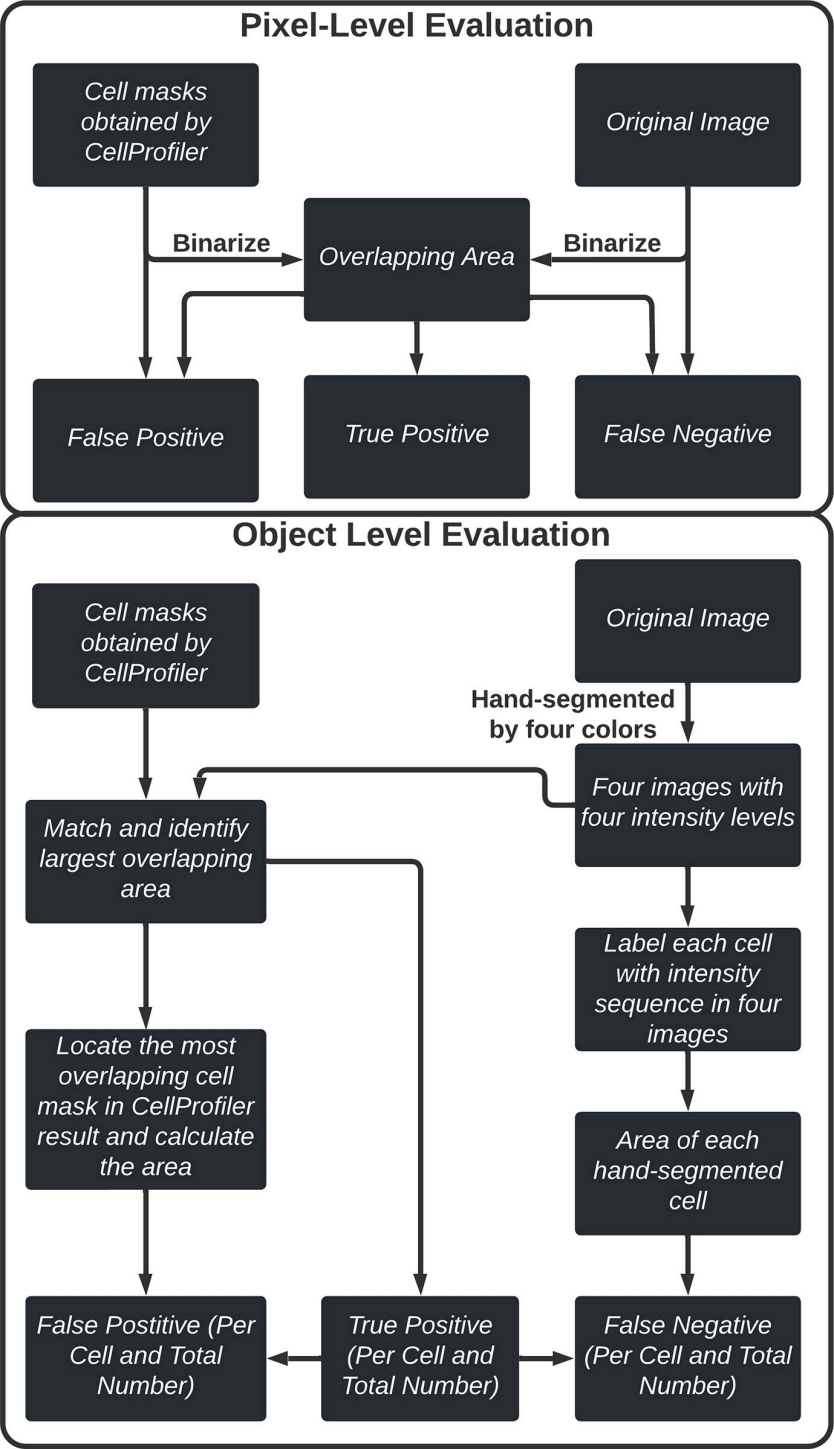
Flow chart of the steps in POSEA to assign pixels as TP, FP, or FN.

The POSEA code computes both traditional pixel-level accuracy metrics [34] and per-object accuracy metrics for the comparison of two segmented images (Fig 1). The first step for POSEA is to load two segmented images, a “test” image and a “ground-truth” image, into the algorithm to compare. The ground truth image contains segmented objects with four unique (non-0) intensity values, and background pixels have a value of 0. The test image contains segmented objects with sequential intensity values from 1 to n, the number of objects in the test image. POSEA computes traditional pixel-level outputs by comparing binary masks of both input images. The masks of the two input images are obtained by changing the intensity of pixels that are above zero to one. Then, each pixel is assigned as TP, FP, and FN based on a comparison of the two images. TP pixels are 1 in both images. FP pixels are 1 in the test image and 0 in the ground truth image. FN pixels are 1 in the ground truth image and 0 in the test image.

#### Object level evaluation

Next, POSEA assigns pixels as TP, FP, or FN at the object level (Fig 1). For robustness and to not require prior knowledge of the object values of the ground truth image, the POSEA algorithm finds the specific intensity values of the objects within the ground truth image by calculating the most frequent non-zero numbers in the array. Then, an image is created for each unique intensity value within the ground truth image. A minimum number of four intensity values ensures the separation of adjacent cells. Each object within the ground truth image is then assigned a unique intensity value, 1 to x’, where x’ is the number of objects in the ground truth image. Similarly, each object in the test image has a unique intensity value from 1 to n’, where n’ is the number of objects in the test image. To calculate pixel assignments, each of the ground truth objects is iteratively evaluated. A loop from 1 to x’ is used to extract each object from the ground truth image and an image mask is generated with the object pixels retaining a value of 1 and the rest of the image set to the background value (0). Next, the object image is multiplied by the test image, and the most frequent pixel intensity in the resulting image is considered to be the matched object from the test image. Once the same object is identified in both the ground truth and test images, the number of TP pixels is calculated. The FP pixels per object is the number of pixels of the object in the test image result minus the number of TP pixels of the object. The FN pixels per object is calculated from the number of pixels of the object in the ground truth image minus the number of TP pixels. To calculate the object level accuracy metrics for the entire image, the sum of the number of TP pixels of each cell is calculated. The FP value is the number of pixels of all objects in the test image result minus the total TP value. The FN value is the number of pixels of all objects in the ground truth image minus the total TP value.

#### Calculations

POSEA calculates Precision (P), Recall (R), and F-measure (F) metrics by both the traditional pixel-level method and by the per-object method. Perfect segmentation results in a value of 1 for Precision, Recall, and F-measure. A value of 0 means no pixels are correctly segmented.


Precision=TruePositives/(TruePositives+FalsePositives)

Recall=TruePositives/(TruePositives+FalseNegatives)

F−measure=(2*Precision*Recall)/(Precision+Recall)

### How to use POSEA

POSEA is based on python 3.7.7. The POSEA code, pseudo code, and example images are posted to GitHub (https://github.com/walshlab/POSEA). POSEA requires two input images, first a ground truth image and second a test image. The background for the ground truth image should be zero. The input images should be grayscale images, with adjacent segmented objects assigned unique integer values. The first outputs of POSEA are the unique intensity levels of the ground truth image. The next outputs are F-measure, Precision, and Recall for a traditional pixel-level evaluation. Then, the final outputs are F-measure, Precision, and Recall metrics for the per-object assessment, computed at the image level. Those three metrics are also calculated for each cell and saved in a CSV document in the python directory.

### Creation and testing of simulated images

#### Validation with binary images

First, POSEA was tested on images with perfectly overlapping objects and objects with no overlap. Two binary 256*256 pixel images were created; one with all pixels having a value of 1, and one image with all pixels having a value of 0. These two images were each assigned as ground truth or test and evaluated by POSEA against itself or the other image for four comparisons.

**Fig 2:**
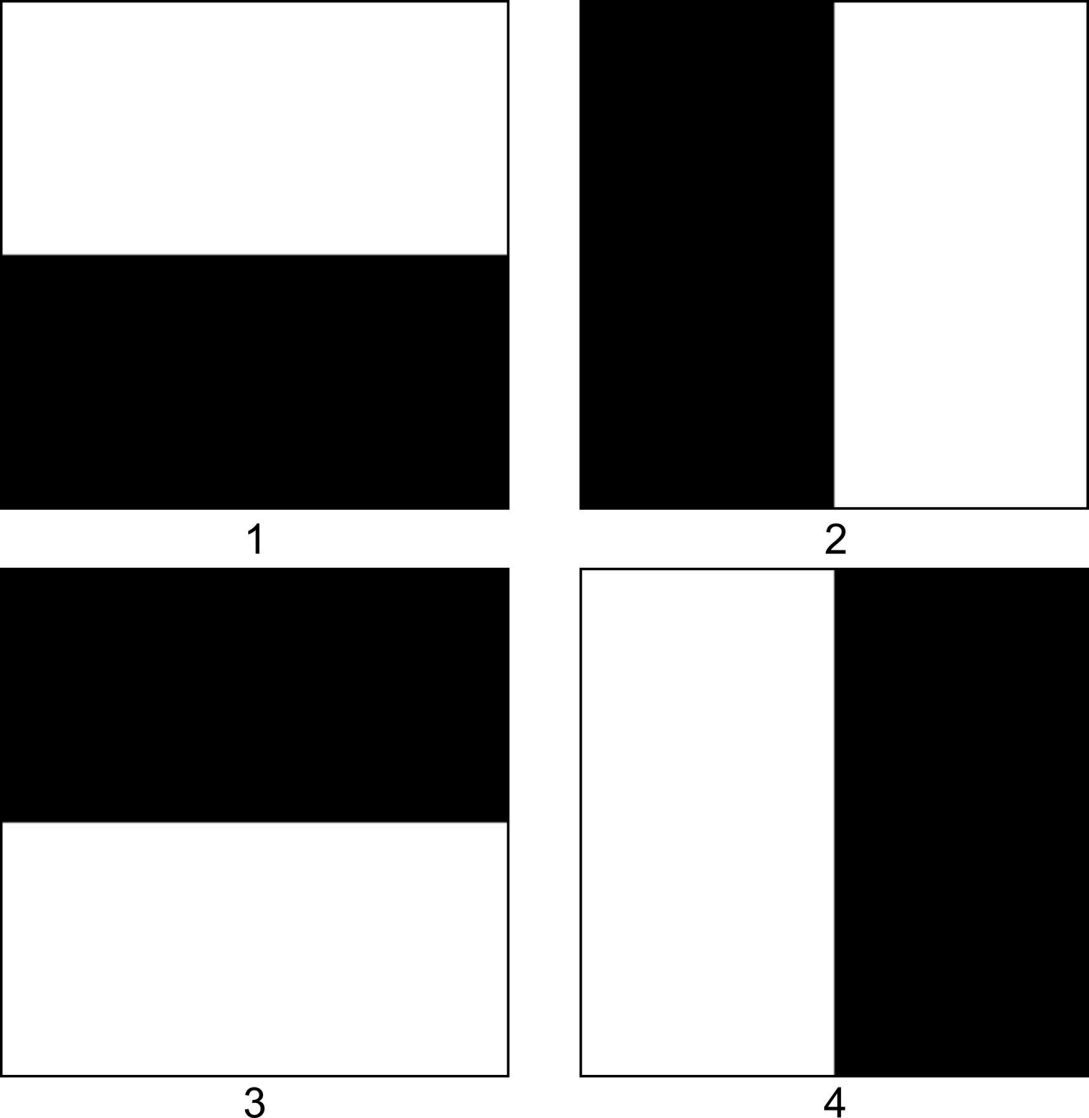
Four half-white (pixel values = 1) and half-black (pixel values = 0) 256*256 pixel images evaluated by POSEA. For each image, the white area was considered the segmented region. Each image was evaluated against the other image, including its replicate.

Next, four half-black (pixel values = 0), half-white (pixel values = 1) binary 256*256 pixel images were created (Fig 2). Iteratively, each image was considered to be the ground truth and evaluated against itself and the three additional images. Therefore, sixteen comparisons were evaluated by POSEA.

### Simulated adjacent cell images

**Fig 3:**
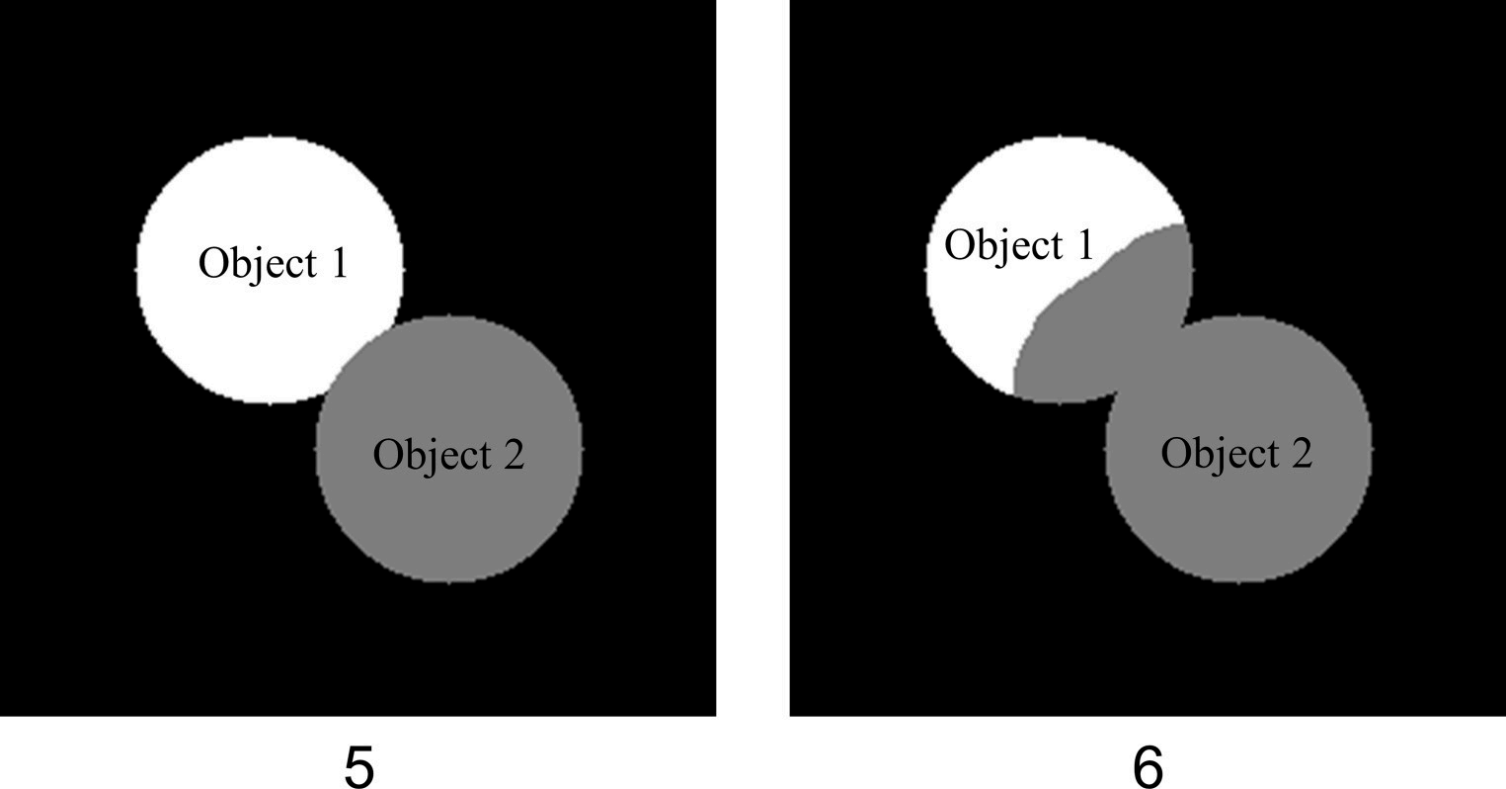
Simulated grayscale images of adjacent objects created to compare the evaluation performance of POSEA and traditional pixel-level analysis for images with pixels misassigned to an adjacent object.

Two 256*256 pixels grayscale images of two adjacent objects of equal size but different intensities were made using OpenCV [40]. The intensities of the two circles are 100 and 200 (Fig 3). POSEA was used to evaluate each image as the ground truth against itself and the other image, respectively. The Precision metric was computed and compared for POSEA and the traditional pixel-level method.

### POSEA evaluation of segmented fluorescence images

#### Fluorescence images of T cells

A previously published [12] dataset of fluorescence images of T cells and paired CellProfiler segmentation results were provided by Drs. Walsh and Skala. The dataset includes about 200 autofluorescence images and matched CellProfiler segmented images of two T cell populations, quiescent T cells and activated T cells. The fluorescence intensity and CellProfiler segmented images are 32-bit grayscale, 256x256 pixel images. Five images of quiescent T cells and five images of activated T cells were selected for manual segmentation of the fluorescence image to create a ground truth image. The images were selected at random to reduce selection bias. The selected quiescent T cell images contained 150–160 cells or unique objects per image while the activated T cell images contained 32–60 cells per image. Quiescent T cells are uniformly sized, round, and separable while activated T cells clump together and are heterogeneous in size, which is more challenging for automated segmentation.

#### Fluorescence images of MCF7 cells

MCF7 breast cancer cells were cultured in high glucose Dulbecco’s Modified Eagle’s Medium (DMEM), supplemented with 1% penicillin: streptomycin, and 10% fetal bovine serum. Cells were plated at a density of 4 x 10^5^ cells per 35 mm glass-bottom imaging dish (MATTEK), 48 hours before imaging. The culture media in the dish was refreshed before imaging.

NAD(P)H fluorescence images were acquired by a customized multi-photon fluorescence microscope (Marianas, 3i) with a 40X water-immersion objective (1.1 NA). A stage top incubator (Okolab) was used during imaging to maintain a physiological environment (37°C, 5% CO_2_, 85% relative humidity). NAD(P)H fluorescence was stimulated by a titanium: sapphire laser (COHERENT, Chameleon) at 750 nm with 18 mW to 20 mW average laser power at the sample. NAD(P)H emission was detected by a photomultiplier tube (HAMAMATSU) coupled with a 550/88 nm bandpass filter. The total time of collecting a 256 x 256-pixel fluorescence lifetime image was around 60 seconds with a pixel dwell time of 50 μs and 5 repeats. Fluorescence lifetime images were integrated across time for fluorescence intensity images.

Five NAD(P)H intensity images of MCF7 cells were randomly selected. The instance segmentation of the cell cytoplasm was generated using a published CellProfiler pipeline [25]. The pipeline first segments cell objects to define the boundaries between cells or cell clumps and background [25]. Then, nuclei regions are identified due to the intensity differences between the nuclei and cytoplasm [25]. Finally, individual cells are identified by propagating out from the nuclei to terminate at either a cell-background boundary or another propagating cell [25]. This process requires the optimization of CellProfiler parameters to match the nucleus size and cell size. Nuclei were identified for diameters of 5 to 25 pixels, a range that encompasses the typical diameters of MCF7 nuclei. Additionally, a threshold correction factor of 0.8 (a number less than 1 alters the threshold value for improved cell boundary identification) and a 30-pixel adaptive window (selected to match the average MCF7 cell size) optimized the performance of the Otsu threshold algorithm for identification of MCF7 cells. Cytoplasm regions were identified by removing nucleus objects from cell objects. Finally, to remove clumped cells and noise, all identified objects were filtered based on the area of the cytoplasm which ranged between 100–500 pixels for MCF7 cells.

#### Segmentation of ground truth images

A ground truth segmented image was created by hand-segmentation of the NAD(P)H intensity images of the cells in ImageJ [41]. Cells were highlighted using the “paintbrush” tool for segmentation. A brush width of 1–5 pixels was chosen based on the need for accurate hand segmentation. Four different intensities were used for the hand segmentation because the four-color map theorem states no more than four colors are required so that no two adjacent regions have the same color [42]. The four intensity values of the segmented ground truth image do not need to be specific values since POSEA automatically recognizes unique intensity values in the image.

#### POSEA evaluation of fluorescence images

POSEA was used to compare the CellProfiler segmented images as test images with the corresponding hand-segmented images as ground truth images. The Precision, Recall, and F-measure output values were recorded. The per-cell data was obtained through the CSV document saved in the python directory.

#### Comparison of POSEA and traditional method

The evaluation metrics F-measure, Precision, and Recall computed by POSEA and the traditional pixel-level method were compared for segmented fluorescence images using R studio [43]. Similarly, R studio was used to build the violin plot for the POSEA per cell data.

#### POSEA evaluation of vehicle images

POSEA was tested on 5 randomly selected synthetic images of cars from a public dataset, ‘Vehicle Rear Side View Synthetic Data Set’ (https://www.kaggle.com/datasets/saratrajput/vehicle-rear-side-view-synthetic-data-set). The dataset contains 5000 8-bit RGB images and corresponding depth images, instance segmentation images, and class segmentation images. The size of each image is 1024*768 pixels. POSEA was used to compare the class segmentation image as the ground truth image with the instance segmentation image, converted to gray-scale, as the test image for 5 randomly selected images.

## Results

### POSEA performance on simulated images

Simulated binary images of completely matching or no matching pixels were evaluated by POSEA to verify the range of the outputs of the algorithm. Images with perfectly overlapping segmented (non-0 intensity) pixel values have an output Precision value of 1 whereas completely different pixel values have an output Precision value of 0. The comparison of an all-black image against an all-black image returns a Precision value of 0, due to POSEA’s assumption that background (non-segmented) pixels have a value of 0.

Then, four half-black, half-white binary images were evaluated by POSEA (Table 1). In these images, the white portion represents a segmented object. The Precision metric is 0 if there is no overlap of objects between the ground truth and test images, 0.5 if half of the pixels overlap between the ground truth and test objects, and 1 if the objects completely overlap in both the ground truth and test images.

**Table 1:** Precision values output from POSEA evaluation of simulated binary images (Fig 2) that are half-white and half-black either horizontally divided (Image 1, Image 3) or vertically divided (Image 2, Image 4).

Precision	Image 1 (ground truth)	Image 2 (ground truth)	Image 3 (ground truth)	Image 4 (ground truth)
Image 1 (test)	1	0.5	0	0.5
Image 2 (test)	0.5	1	0.5	0
Image 3 (test)	0	0.5	1	0.5
Image 4 (test)	0.5	0	0.5	1

Two simulated grayscale images of adjacent objects (Fig 3) were evaluated by POSEA (Tables 2 and 3). The Precision value for the comparison of these two images by the traditional pixel-based method is 1. POSEA calculates values of 1 for Precision, Recall, and F-measure for each object in Image 5 and Image 6 when the same image is used as both the ground truth and test image. When Image 5 is evaluated with Image 6 as the ground truth image, for Object 1, the Precision, Recall, and F-measure values are 0.65, 1, 0.79, respectively. For Object 2, the Precision, Recall, and F-measure values are 1, 0.75, and 0.85, respectively. When Image 6 is evaluated with Image 5 as the ground truth image, for Object 1, the Precision, Recall, and F-measure values are 1, 0.65, and 0.79, respectively. For Object 2, the Precision, Recall, and F-measure values are 0.75, 1, and 0.85, respectively.

**Table 2:** Precision values output from the traditional pixel-level evaluation method of the simulated grayscale images of adjacent cells (Fig 3).

Traditional Method	Image 5 (test)	Image 6 (test)
Image 5 (ground truth)	1	1
Image 6 (ground truth)	1	1

**Table 3:** Precision, Recall, F-measure values output from POSEA per cell evaluation of the simulated grayscale images of adjacent cells (Fig 3).

POSEA Method	Object	Image 5 (test)	Image 6 (test)
Image 5 (ground truth)	Object1	Precision: 1 Recall: 1 F-measure: 1	Precision: 1 Recall: 0.65 F-measure: 0.79
Image 5 (ground truth)	Object2	Precision: 1 Recall: 1 F-measure: 1	Precision: 0.75 Recall: 1 F-measure: 0.85
Image 6 (ground truth)	Object1	Precision: 0.65 Recall: 1 F-measure: 0.79	Precision: 1 Recall: 1 F-measure: 1
Image 6 (ground truth)	Object2	Precision: 1 Recall: 0.75 F-measure: 0.85	Precision: 1 Recall: 1 F-measure: 1

#### Evaluation of segmented autofluorescence images

**Fig 4:**
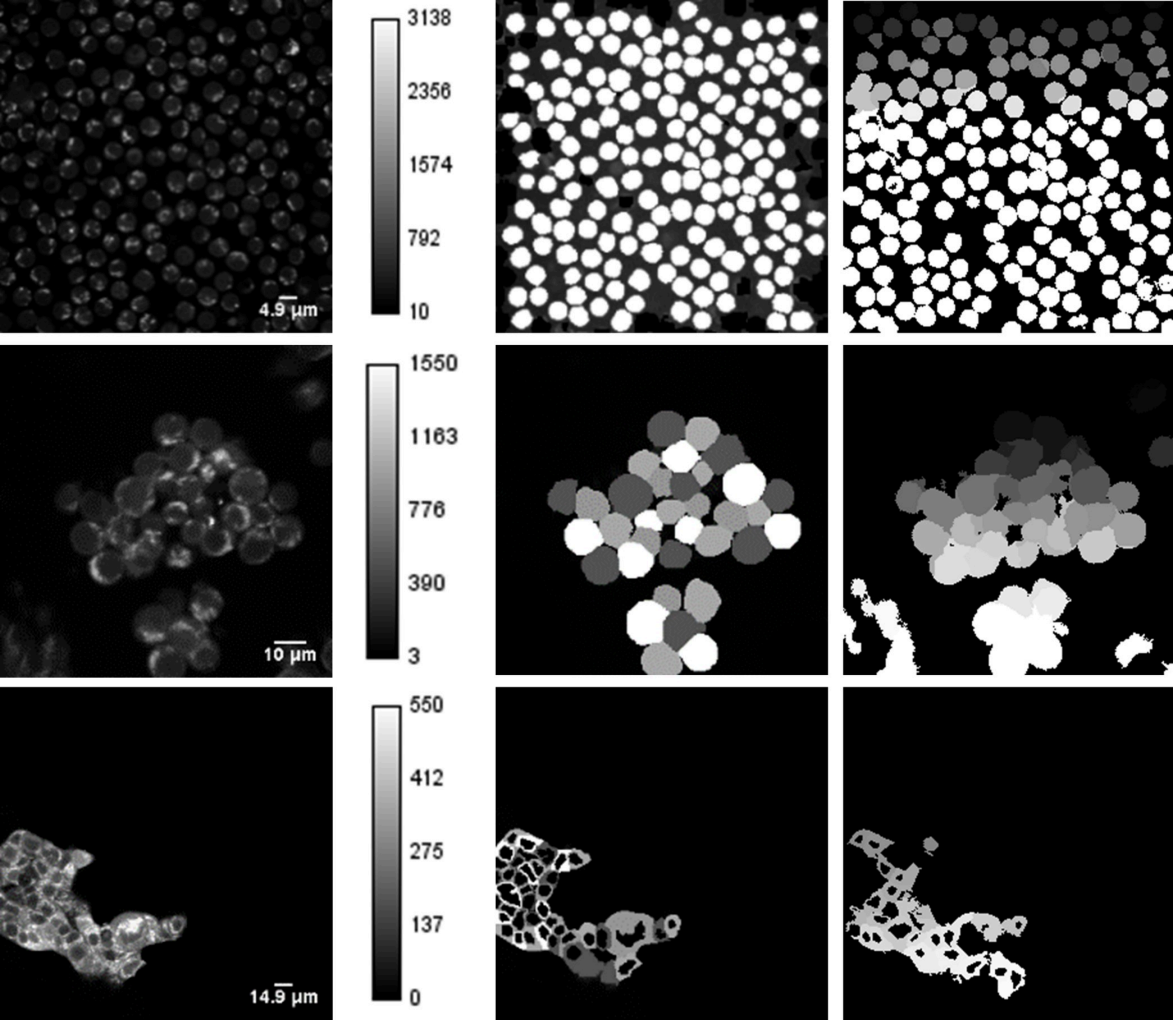
Representative fluorescence (first column) images, ground truth segmented images (second column), and CellProfiler segmented images (third column) of quiescent T cells (first row), activated T cells (second row), and MCF7 cells (third row). Quiescent and activated T cells are segmented into individual cells whereas MCF7 cells are segmented into the cytoplasm.

Within cells, NAD(P)H is primarily localized to the cytosol and mitochondria. Therefore, cells in autofluorescence images exhibit high intensity in the cytosol while the nucleus remains dim (Fig 4). Representative fluorescence and segmentation images of quiescent T cells, activated T cells, and MCF7 cells show the differences in cell shape and clustering among the groups (Fig 4).

**Fig 5:**
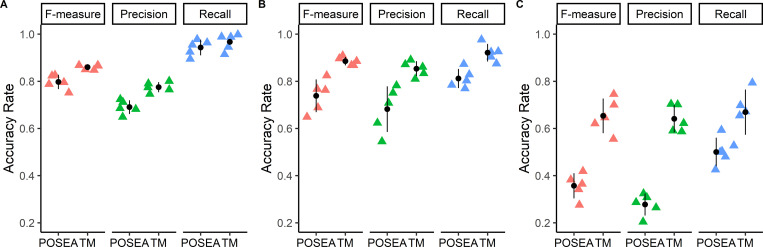
F-measure, Precision, and Recall values calculated by POSEA and the traditional pixel-level method (TM) to compare CellProfiler segmentation results with hand-segmented, ground truth images for quiescent T cells (A), activated T cells (B), and MCF7 cells (C). Each colored triangle is the value for an image (n = 5 images per group). The black circle and lines represent the mean and standard deviation of the 5 data points in each group.

POSEA outputs for image-level F-measure, Precision, and Recall metrics were compared with traditional pixel-level metrics (Fig 5). F-measure, Precision, and Recall values computed by the traditional pixel-level method are higher than the corresponding metric computed by POSEA for each cell type (Fig 5). The POSEA output metrics of F-measure, Precision, and Recall are greatest for the segmentation results of quiescent T cells and lowest for the MCF7 cells. For the traditional pixel-level evaluation method, F-measure, Precision, and Recall values of activated T cells are slightly greater than the metrics of quiescent T cells. The MCF7 cells have the lowest evaluation metrics by the traditional pixel-level analysis, however, the F-measure, Precision, and Recall values are higher than 0.6.

### Evaluation results per cell using POSEA

**Fig 6:**
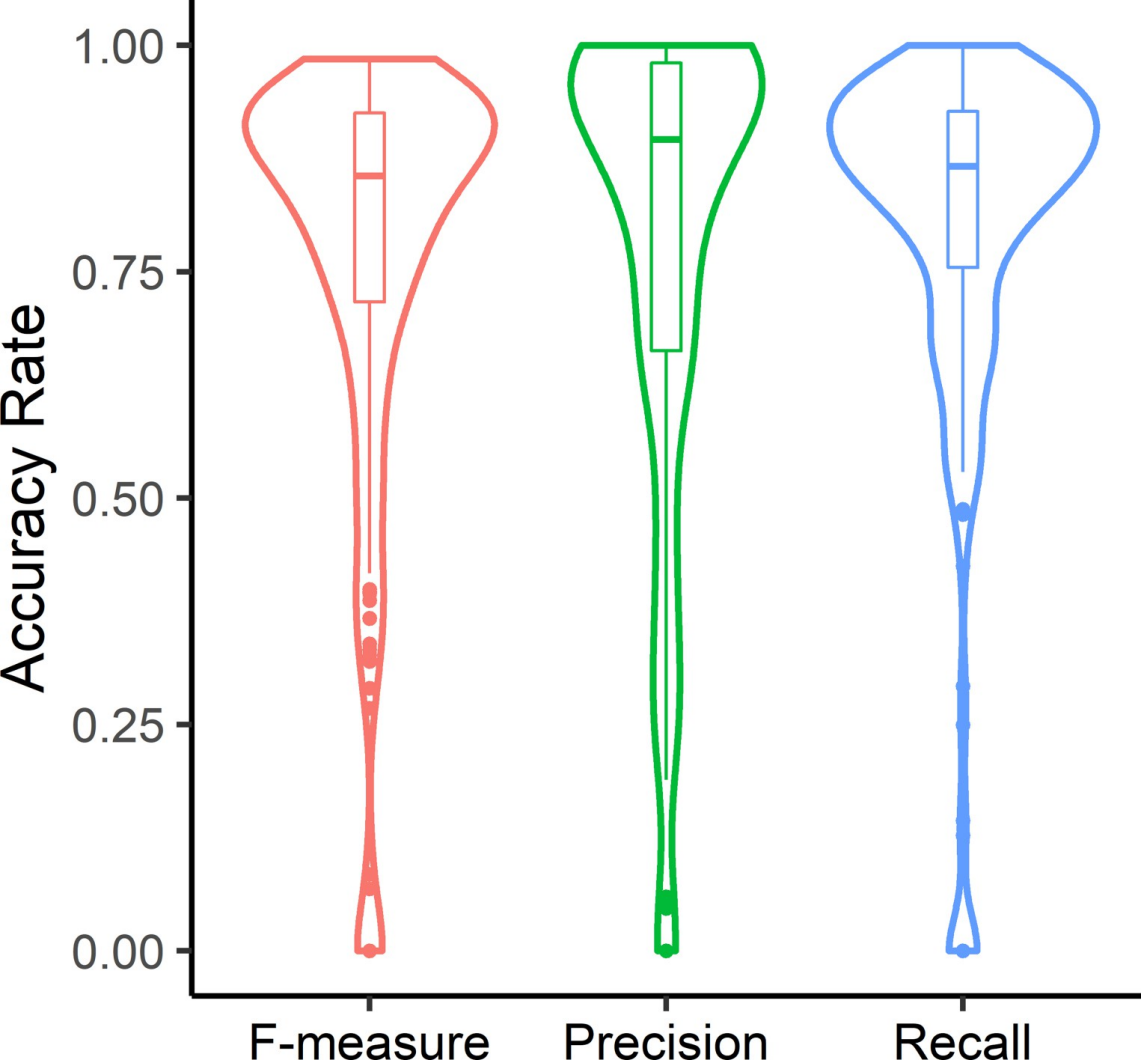
Violin plots and boxplots showing the distribution of the F-measure, Precision, and Recall values calculated by POSEA for each segmented object within the activated T cell images (n = 225 cells from 5 images).

POSEA evaluation metrics for each object within the activated T cell images were analyzed to compare the CellProfiler segmented image with the hand-segmented ground truth image (Fig 6). At the object level for activated T cells, Precision is the highest mean value and Recall is the lowest value. The histograms of each evaluation metric have a skewed distribution of low values.

### POSEA time consumption

The time required for POSEA to evaluate each image was measured (Table 4) using a desktop with an Intel i9-9900KF CPU and an NVIDIA GeForce RTX 3080 GPU.

**Table 4:** Average (n = 5 images) time consumption of POSEA and the traditional pixel-level method to compare ground truth and segmented images of Quiescent T cells, Activated T cells, and MCF7 cells. The average number of cells per image is 154 for Quiescent T cells, 45 for Activated T cells, and 44 for MCF7 cells.

Average time (s)	Quiescent T cells	Activated T cells	MCF7 cells
Traditional Method	0.056	0.066	0.022
POSEA	2.047	0.406	0.413

### Evaluation results of the vehicle dataset using POSEA

Average (n = 5 images; 15 objects) F-measure, Recall, and Precision values calculated by POSEA are 0.9691, 1, 0.9843. The traditional method matches POSEA at the image level accuracy as there are no pixels misassigned to adjacent objects. For the object level analysis, the average (standard deviation) F-measure, Recall, and Precision values are 0.9860 (0.009744), 1 (0), and 0.9725 (0.018951), respectively.

## Discussion

Here, an object-based supervised evaluation algorithm (POSEA) is demonstrated for accurate assessment of segmented images with a large number of unique, adjacent objects. POSEA was tested on simulated images of increasing segmentation complexity to demonstrate the accuracy and performance of POSEA. Using autofluorescence images of quiescent T cells, activated T cells, and breast cancer cells, segmented by an automated CellProfiler pipeline, the differences between per-object evaluation and a traditional pixel-level evaluation method were demonstrated. Finally, the unique ability of POSEA to calculate segmentation performance at an object level was shown for autofluorescence images of activated T cells.

POSEA was rigorously tested on simulated images of increasing complexity to define the performance of the algorithm. POSEA Precision outputs for matching non-0 pixel intensities or all mismatching pixel values are the expected values of 1 or 0, respectively. Due to the POSEA assumption that pixel values of 0 are background and these pixels do not belong to a segmented object, the output precision value of a black (intensity value = 0) image evaluated against itself is 0. Likewise, POSEA calculates the expected output Precision values for half-white and half-black images: when 50% of pixels match, the Precision value is 0.5 (Table 1). Finally, images of two adjacent objects, with the same outer perimeter for the object cluster but different individual objects (Fig 3), were simulated to directly compare the performance of POSEA with the traditional pixel-level method. The traditional pixel-level method resulted in inaccurate Precision values of 1 (Table 2). In contrast, POSEA calculated accurate Precision, Recall, and F-measure metrics for both objects, accounting for the pixels incorrectly assigned to the adjacent object. Although POSEA outputs Precision and Recall values of 1 sometimes when non-identical objects are compared, this is due to the formulas for calculations of Precision, which only includes TP and FP, and Recall, which only includes TP and FN. Altogether the simulated image experiments define the range of output values for POSEA and demonstrate the advantage of POSEA for computing segmentation accuracy of adjacent objects. Since the accuracy metrics are calculated by matching each object in the ground truth images and the corresponding objects in the segmentation results, the performance of POSEA is immune to the number of objects, object size and spatial distribution.

POSEA-computed Precision, Recall, and F-measure values are more accurate than traditional pixel-level evaluation of fluorescence images of three different cell types, quiescent T cells, activated T cells, and breast cancer cells, MCF7. The POSEA Recall, Precision, and F-measure metrics are lower than the metrics computed by traditional mask assessment due to the inclusion of the per-object accuracy by POSEA (Table 2). Lower metrics are expected for POSEA due to pixels incorrectly assigned to adjacent objects, as is demonstrated with the simulated objects in Images 5 and 6 (Fig 3, Tables 2 and 3). POSEA, but not the traditional pixel-level method, computed higher values for Precision, Recall, and F-measure of the CellProfiler segmentation of quiescent T cells than activated T cells (Fig 5), as is expected since the quiescent T cells are easier for automated segmentation, due to their isolated and round shapes (Fig 4). POSEA is not limited to specific output metrics but can calculate multiple metrics based on TP, FP, FN which are the basic metrics for evaluation algorithms [44]. Recall, Precision, and F-measure metrics were chosen since they are sensitive to under-segmentation and over-segmentation [45]. The POSEA results for instance and class segmented automobile images demonstrate its robustness and transference across image types and datasets.

POSEA uses ground truth objects to match segmentation results, which allows the computation of the per-object evaluation metrics. Iteration by object allows the calculation of pixel classifications as TP, FP, and FN for each cell. These per-object metrics allow the identification of poorly segmented objects. Therefore, the per-object metrics are unique to POSEA and provide an advantage over other evaluation methods that output metrics only at the image level [26,28,46]. For example, as shown in Fig 6, activated T cells segmented by CellProfiler generally have high Precision values (>0.8), yet a number of cells have low Precision values. Objects with low Precision values have a high FP rate which suggests under-segmentation. To visualize and further investigate why those cells have low Precision values, a Precision threshold could be set to display the locations of the cells that were poorly segmented and visual inspection could provide information on why the segmentation protocol failed for those particular cells. Interpretation of the POSEA per-object evaluation metrics can be used to inform strategies to improve segmentation.

POSEA is a robust and easily implemented tool for segmentation evaluation. However, POSEA also has some limitations. First, POSEA requires grayscale images for which the intensity values correspond to unique objects. Next, as a supervised evaluation method, POSEA requires a reference or ground truth image. Hand-segmentation to generate a ground truth image can be time-consuming for a large number of images or a large number of objects [35]. POSEA is slower than the traditional method (Table 4), because of the iterative nature to compute the accuracy of each object. The quiescent T cell images required a longer time than the quiescent and cancer cell images because of the greater number of cells in the quiescent T cell images. Currently, POSEA can only analyze one object class at one time, for example, the cell mask or cytoplasm. If the segmentation output includes multiple object classes, POSEA would have to evaluate them separately.

In summary, POSEA provides accuracy metrics combining object and pixel-level assessments of segmented images with a large number of unique, adjacent objects. Therefore, POSEA is a useful tool for the optimization of instance segmentation methods for applications that require high pixel-level performance for the segmentation of adjacent objects, as is necessary for cell segmentation within microscopy images. Evaluation of segmented microscopy images demonstrates that POSEA is more accurate than traditional pixel-level evaluation for images with a large number of adjacent objects. Moreover, POSEA provides segmentation accuracy metrics for each object for the identification of the poorly-segmented objects in an image. POSEA is not limited in application to microscopy images of cells but can be applied to evaluate any pair of segmented images to compare segmentation methods and optimize automated segmentation techniques.
